# The Effects of Biofeedback on Performance and Technique of the Boxing Jab

**DOI:** 10.1177/00315125211013251

**Published:** 2021-05-03

**Authors:** Mark A. Chen, K. Spanton, P. van Schaik, I. Spears, D. Eaves

**Affiliations:** 1 University of Teesside, Department of Science, Middlesbrough, UK; 2 Newcastle University, Population Health Sciences Institute, Newcastle, UK

**Keywords:** attention/distraction, auditory measurement, human performance, martial arts, sport/athletic technique

## Abstract

A growing body of research has addressed the application of movement-based biofeedback techniques for improving sports performers’ gross motor skills. Unlike in previous research, we aimed in this study to quantify the effects of this “external” biofeedback on selected performance and technique variables for the boxing jab among both novices and experts. The technical setup included two inertial measurement units linked wirelessly to a video game system with audio output. The units were configured to provide auditory external biofeedback, based on the peak acceleration of the bag (i.e., biofeedback with an external attentional focus). Sixteen participants (8 novices and 8 experts) performed boxing jabs against the bag in blocked phases of biofeedback. When compared to baseline, the acute effects of externally focused biofeedback on peak bag acceleration were possibly positive in both retention phases for novices (d = 0.29; d = 0.41) and likely positive for experts (d = 0.41; d = 0.30), respectively. The experts’ performance improvements were accompanied by substantive increases in trunk rotation, though this was not true for the novices. Thus, technique improvements can be promoted indirectly via externally focused biofeedback, but only when these actions are within the performers’ motor repertoire. Overall, biofeedback via inertial sensors appears to be a potent technique for modifying human movement patterns in both experts and novices. This low-cost technology could be used to support training across sports, rehabilitation and human-computer interactions.

## Introduction 

In human movement-related literature there is a general consensus that refinements in movement technique are associated with improved motor performance (Carson et al., 2016). There is, however, a recognized shortage of feedback interventions that use quantitative movement data to inform and improve *both* motor performance and technique. Although in its relative infancy, movement-related, externally focused biofeedback has been used to improve fine motor skills for computer tracking tasks (Jarus et al., 2015) and key pressing tasks (Rossettini et al., 2017) and to improve gross motor skills such as marching among British army recruits (Sharma et al., 2014). In particular, Eaves et al. (2011) showed that biofeedback relating to *distal*, rather than *proximal* features of the action facilitated the acquisition of complex dance moves among novice performers. It is not clear from this literature how biofeedback might improve expert performance.

Inertial measurement units offer new opportunities for providing movement-related biofeedback. These low-cost units – typically consisting of a tri-axial accelerometer, gyroscope and magnetometer – have been increasingly employed in recent movement-related research, and they have been able to capture movement variables from a specific body segment (e.g., the trunk) or an object to which these body segments are attached (e.g., tennis racket). The acceleration and angular velocity data from these units, which are challenging to derive using traditional vision-based motion capture systems, may be pertinent to the requirements of movement-related biofeedback systems, as accelerations are closely related to the forces that generate movements (i.e., muscle activity) and cause body damage (e.g., joint stress). Examples of inertial measurement units in biofeedback applications include devices to (a) enable recreational runners to adjust their patterns of body movements, thus reducing the shock and risk of injury during landing (Crowell et al., 2011), and (b) enable knee osteoarthritis sufferers to reduce medial pressures on the tibia condyle (Barrios et al., 2010).

The above studies have obtained performance improvements by providing biofeedback about movement kinematics, but the performance-related feature highlighted with this feedback, while crucial, has often been overlooked. To this end a substantial body of new research has emerged, showing that task instructions designed to promote an external versus internal focus of attention (i.e., directing attention toward the movement outcome rather than form) can improve force production across a range of tasks (see, for a review, Marchant, 2011). Examples include athletes’ standing long jump distance (Porter et al., 2012), vertical jump height (Wulf & Dufek, 2009), discus throwing (Zarghami et al., 2012) and elbow flexion (Marchant et al., 2009). However, not all differences are explained by force in these tasks. Although Wulf and Dufek (2009) found that physically active students generated a significantly larger vertical impulse during a vertical jump when focusing externally opposed to internally, Ducharme et al. (2016) found that, for a similar population, greater distances jumped in the external condition were not explained by differences in peak force. Overall, these findings are broadly in line with the theory of event coding (Hommel et al., 2001), positing that actions are essentially planned in terms of their anticipated sensory consequences. Therefore, directing a performer’s attention to the more distal features of their movement (i.e., the impact of movement upon the environment) can serve to automate processes of motor planning and execution and gain advantages in force production and execution (Vance et al., 2004).

Previous research suggests that both experts and novices benefit more from an external than an internal focus of attention (e.g., Wulf & Su, 2007). For example, Ille et al. (2013) showed that both experts and novices improved their reaction times and 10 m sprint times when focused externally, compared to internally. However, these conclusions do not consider, first, that an external focus may be moderated by expertise and second, that these differences are rarely investigated in terms of key biomechanical factors (e.g., Winkelman et al., 2017; Wulf & Dufek, 2009). Winkelman et al. (2017) found faster sprint times for soccer players but not experienced sprinters for external versus internal focus. In addition, the sprinters showed no differences in kinetic variables between conditions, thus supporting the findings of Ducharme et al. (2016). In the only study using combat sports techniques, Roseman (2017) investigated taekwondo kicking among novice and expert martial artists and found that force-accuracy was higher in the distal external condition compared to the proximal external and internal conditions, suggesting that focusing further away from the body is associated with improved results compared to focusing near or on the body. Therefore, in the present study, we developed a novel biofeedback technique to promote an external focus of attention on a force production task. Here we employed a more in-depth analysis of the biomechanical variables underlying movement outcome for both experts and novices than has previously been undertaken.

While previous studies have generally used verbal instructions to manipulate external attentional focus, few studies have investigated the biomechanical factors that underpin the performance changes. We were interested in how expert and novice performers would respond to a computer-generated audio cue that provided externally focused biofeedback about the punch force of a boxing jab. We assessed both the overall force produced using a boxing jab, and the kinematics of the jab technique. The jab requires rapid and complex motor skills, involving a series of coordinated and forceful muscular contractions (Lenetsky et al., 2020). Stanley et al. (2018) found that the jab exhibited a proximal-distal sequence for the shoulder and elbow joints, respectively, with the shoulder reaching peak angular joint velocity approximately 12% before the elbow during jab execution. In similar high-speed sporting movements, such as the tennis serve or the golf drive, the proximal movement segments are known to reach their maximal angular velocities prior to the distal movement segments, a process believed to facilitate effective transmission of joint torques between segments (Putnam, 1991). Presumably the ability to exploit and maximize this process underlies experience-related advantages among experts (c.f., Konczak et al., 2009). The main aim of the present study was to examine whether this externally focused biofeedback could improve motor learning in *both* experts and novices. Therefore, while both the jab technique and the force produced were expected to vary between expert and novice boxers, we examined whether external biofeedback about the punching force would modulate both the outcome and technique for both participant groups.

## Method

### Participants

Sixteen males participated in this study (8 experts with a mean boxing experience of 5.1 years (*M*_age_ = 24.1, *SD* = 4.5 years; *M*_weight_ = 79.3, *SD* = 17.0 kg; *M*_height_ = 178.0, *SD* = 5.7 cm) and 8 novices with no boxing experience (*M*_age_ = 21.6, *SD* = 2.8 years; *M*_weight_ = 77.3, *SD* = 4.3 kg; *M*_height_ = 176.1, *SD* = 12.6 cm). The experts were regional level amateur boxers with at least one bout within the preceding six months of data collection. This is similar criteria to previous studies that have used experienced boxers (e.g., Lenetsky et al., 2020)

All participants completed an informed consent form prior to engagement in the study, and they completed a medical questionnaire to exclude any medical condition that would preclude their participation. The questionnaire highlighted any participants with cardio-vascular, heart or breathing related conditions that would exclude them from the study. The study was approved by the local university research ethics committee (Res/E/1568).

### Design

The between-subjects factor was skill level (expert or novice), and the within-subjects factor was performance block. The dependent variables were peak bag acceleration (m.s^–2^) on impact and peak angular velocity (rad.s^–1^) of the scapula. Novices performed 10 initial jabs (Block 1) with no feedback for familiarization. Subsequently, both groups performed 20 baseline jabs (Block 2) without auditory feedback. Blocks 3–7 were the learning phase, during which both groups performed 20 jabs each, with half the trials in each block randomly ordered to provide auditory feedback. The volume of the white noise for biofeedback was based only on the bag acceleration. The participants then undertook the retention phase of three blocks (Blocks 8–10), during which they performed 20 jabs using a retention-transfer-retention (A-B-A) sequence (Poolton et al., 2006).

### Procedure

#### Initial Instructions

**Figure 1.**
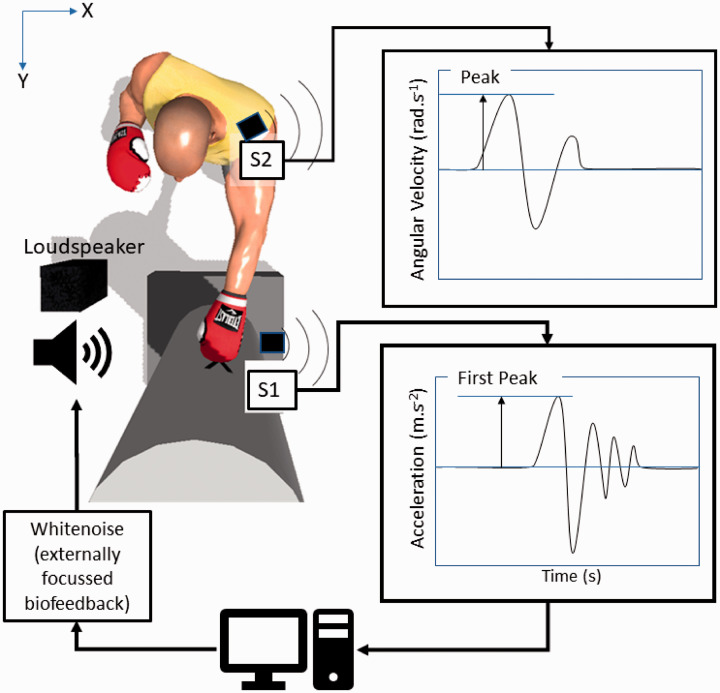
Birds-Eye View of the Equipment Used to Collect Data (S1 and S2) and Generate Biofeedback (S1) in the Form of White Noise. Angular velocity from S2 was taken about the Z-axis (i.e. vertical direction) and bag acceleration from S1 was taken along the Y-axis (i.e. anterior-posterior direction). Data processing was automated with the time from trial completion to feedback being less than 30 ms.

For the experimental task, all participants adopted a standardized boxing stance (see Figure 1), wherein they stood, bare-chested, at arm’s length away from a stationary punch bag. They were then required to make a left-handed boxing jab against the bag. They stood with their left leg forward and their right foot slightly behind, a shoulder width apart. Novice participants were given a pre-test familiarization session, during their first session, to introduce the correct technique.

#### Pre-test

After receiving standardized verbal instructions, novice participants completed 30 jab trials, with instructions to raise the left heel slightly at impact, rotate the left hip towards the bag, move the body weight onto the left front foot during the jab, tuck the left elbow in as long as possible, rotate the pectoral girdle towards the bag making it a long jab, fully extend the elbow during impact, and rotate the fist downwards during impact. Experts did not take part in the pre-test session, as they were experienced boxers.

#### Familiarization and Baseline Phase (Blocks 1–2)

All boxers performed 10 jabs in Block 1 to reduce variability in the dependent variables. Subsequently, all participants were given two minutes rest in order to reduce potential fatigue effects before starting the baseline trials (Block 2). These trials required both experts and novices to complete 20 jabs without auditory feedback. Participants then had a 5-minute rest before starting the blocked feedback sessions (Blocks 3–7). Blocks 3–7 involved 20 jabs, with two minutes rest between blocks with half the trials within each block randomly ordered to provide auditory feedback.

#### Learning Phase (Blocks 3–7)

At the start of each block participants were told that the volume of the white noise (biofeedback) administered after each jab was proportional to the first peak in bag acceleration detected immediately after impact. Before each jab, the researcher counted down “3, 2, 1” to provide the cue to initiate the jab. White-noise feedback was randomly ordered and presented at the end of the jab for 10 out of the 20 trials. The time lag between the end of the punch and the biofeedback was <30 ms. In each trial, participants were instructed to aim their punch at the ‘X’ target on the bag as hard as possible and to use the white noise feedback to assess and monitor their own performance. Note that, at the same time, the sound volume that was delivered was calculated based on bag displacement. A white-noise sound was used as it has been found that auditory feedback can improve timing and motor performance, both of which are crucial features of jab movement (Lai et al., 2002). The feedback was given on only half of the trials in a randomly selected order to prevent the performers from becoming over-reliant on the feedback. Moreover, reduced feedback schedules are well known to aid retention of the motor skill, since these can promote better intrinsic error detection and correction processes (Anderson et al., 2005).

#### Retention Phase (Blocks 8–10)

After Block 7 the participants were deliberately distracted to prevent them from thinking about the task (Poolton et al., 2006) by completing the Reinvestment Scale (Masters et al., 1993) and the Big 5 Personality-44 inventory (Kaiseler et al., 2012). The participants then undertook three blocks of 20 jabs (Blocks 8–10) using a retention-transfer-retention (A-B-A) sequence (Poolton et al., 2006). The purpose of Blocks 8–10 was to provide an assessment of retention (Blocks 8 and 10) on either side of transfer (with dual tasking, Block 9). Blocks 8 and 10 tested jab performance without feedback. Block 9 tested transfer under a condition of increased working-memory load. The dual task involved listening to a pre-recorded track of high and low pitches. Participants counted the high pitches and shouted out the cumulative number for each count in the sequence before waiting for the next cue to jab. A two-minute rest period was given between all blocks. The purpose of this A-B-A design was to quantify performance during blocks where biofeedback was administered relative to performance in the absence of feedback and during interference.

### Equipment, Materials, and Measures

The stationary punch bag (height 1.8 m, diameter 0.5 m, Lonsdale, UK) was fitted with an adhesive target at height 1.54 m (marked as 'X' on Figure 1). An inertial measurement unit (S1, Figure 1) was comprised of a triaxial accelerometer capable of measuring accelerations of ±78 m.s^–2^ (LIS331DLH, STMicroElectronics, USA) and was mounted on the base of the bag in order to measure the acceleration due to the jab. A second inertial measurement unit (S2, Figure 1) was a ±2000 degrees^–1^ triaxial gyroscope (ITG3200, Invensense, USA) that was placed on the acromion process of the left shoulder in order to quantify angular velocity of the upper torso during jab-throwing. Data from the units were transmitted (180 Hz) by radio frequency to a paired receiver unit housed within a USB dongle attached to a desktop computer. The received data were read using a commercially available games engine (Unity3D, USA). The data were processed in order to derive the first peak of acceleration immediately after impact from the base-mounted sensor (S1, Figure 1) and the peak of angular velocity of the upper torso from the pectoral acromion process mounted sensor (S2, Figure 1.) Bag acceleration (m.s^–2^) and angular velocity (rads^–1^) of the trunk were measured (Figure 1) for all trials including baseline. In trials where feedback was delivered, the acceleration data from the bag-mounted sensor (S1) was processed to deliver white noise via the loudspeaker. The volume (V) of the white noise was determined by the following equation:

$$\begin{array}{l} \text{V}=\text{12}0\times \left(\text{peak}\;\text{value}\;\text{of}\;\text{acceleration}\;\text{in}\;\text{the}\;\text{current}\;\text{trial}\right)\\ /\left(\text{peak}\;\text{value}\;\text{of}\;\text{acceleration}\;\text{recorded}\;\text{at}\;\text{baseline}\right) \end{array}$$

### Data Analysis

Difference scores for both participant groups were calculated between baseline performance (Block 2) and performance on each of Blocks 8 (Retention 1), 9 (Dual task) and 10 (Retention 2). Data were log transformed and then back transformed to obtain the difference between Block 2 (baseline) and Blocks 8, 9 and 10. Magnitude-based inferences informed us as to whether the intervention effect would be positive, trivial or negative in the population (Batterham & Hopkins, 2006). Percent differences, with uncertainty of the estimates shown as 90%-confidence intervals for the differences in bag acceleration and angular velocity between and within Block 2 and Blocks 8,9 and 10 were calculated using a customized spreadsheet (Hopkins, 2007).

## Results

**Figure 2.**
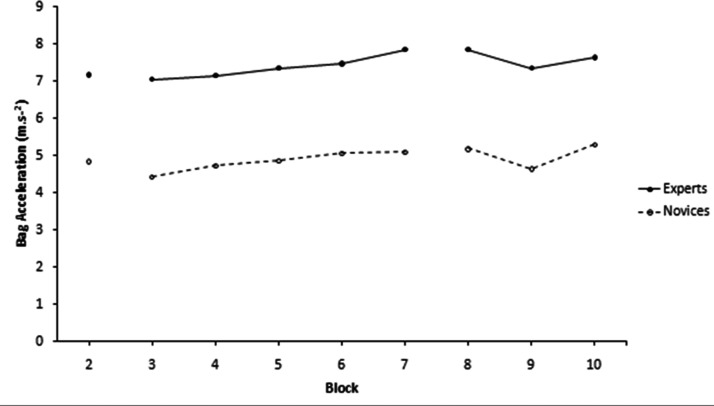
Shows Bag Acceleration (m.s^–2^) From Baseline to Block 10.

**Figure 3.**
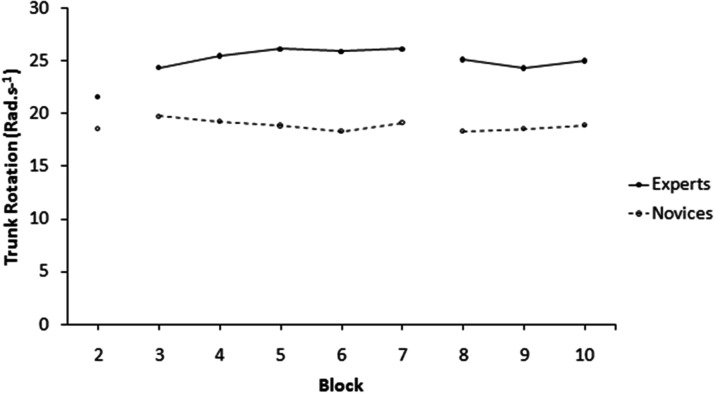
Shows Angular Velocity (Rad.s^–1^) From Baseline to Block 10.

According to descriptive data presented in Table 1, novice participants’ peak bag acceleration increased in the retention phases from baseline; peak bag acceleration decreased in the dual task phase from baseline. Among experts, peak bag acceleration increased in both the retention and dual task phases. For novices’ angular velocity, there were increases from Retention 1 to Retention 2, relative to baseline, while experts’ angular velocity remained similar throughout the final three blocks. Regarding the leaning blocks, for bag acceleration (Blocks 3–7) there were no differences between the groups, as the slight upward trend was the same for both groups. For angular velocity, there was a slight difference in trend between the groups, with the experts slightly increasing over Blocks 3–5 before going constant over Blocks 6–7, compared to the novices slightly decreasing over Blocks 3–5 before going constant over Blocks 6–7 (see Figures 2 and 3).

**Table 1.** Mean (SD) Peak Bag Acceleration and Angular Velocity by Experience and Block.

	Novice		Expert	
	Mean	*SD*	Mean	*SD*
Bag acceleration
Baseline	4.85	(0.97)	7.16	(1.61)
Retention 1 (Block 8)	5.18^a^	(0.99)	7.85^b^	(1.51)
Transfer (Block 9)	4.65	(0.75)	7.35	(1.37)
Retention 2 (Block 10)	5.29^a^	(0.86)	7.64^b^	(1.41)
Angular velocity				
Baseline	18.59	(4.49)	21.56	(4.11)
Retention 1 (Block 8)	18.29	(4.71)	25.13^c^	(4.74)
Transfer (Block 9)	18.50	(5.12)	24.31	(4.97)
Retention 2 (Block 10)	18.86	(4.06)	25^a^	(4.56)

^a^Possibly positive effect.

^b^Likely positive effect.

^c^Very likely moderate effect.

In order to establish that our selection of the two groups resulted in the skill differences we expected and that our measures were sensitive, we compared these samples on both measures at baseline with magnitude-based inference. In this analysis and in those following, the smallest important effect size was defined as *d* = 0.2. The effect of expertise on peak bag acceleration was most likely positive (with chances of positivity/triviality/negativity at 99.9%/0.1%/0.0). The effect of expertise on peak angular velocity was likely positive (with chances of positivity/triviality/negativity at 94.9%/3.9%/1.2).

Regarding peak angular velocity (see Table 2), novices showed a likely trivial effect of auditory biofeedback on the difference in peak angular velocity from baseline in Retention 1 and a possible trivial effect in Retention 2. The experts showed a very likely moderate and likely positive effect in Retention 1 and Retention 2, respectively. For novices, the velocity difference from baseline was likely trivial in dual tasking. For the experts, the small difference in dual tasking was likely positive.

**Table 2.** Magnitude-Based Inference Statistics Comparing Baseline With Retention and Transfer Blocks.

	Raw difference in means compared with baseline	% difference in means	Effect size	Probability of benefit/negligible/harm
Novices				
PBA R1	0.33 ± 0.69	6.88 ± 14.45	0.29	61/31/8
PBA TR	–0.20 ± 0.53	–3.46 ± 9.72	–0.16	9/49/43
PBA R2	0.44 ± 0.74	9.67 ± 15.11	0.41	73/22/5
PAV R1	–15.46 ± 79.48	–1.78 ± 6.55	–0.06	3/84/13
PAV TR	–3.20 ± 94.47	–0.78 ± 7.36	–0.03	6/83/11
PAV R2	17.03 ± 137.86	2.50 ± 15.03	0.08	33/52/15
Experts
PBA R1	0.69 ± 0.49	10.28 ± 7.54	0.41	90/10/0
PBA TR	0.19 ± 0.48	3.28 ± 6.73	0.13	33/64/3
PBA R2	0.48 ± 0.44	7.35 ± 6.58	0.30	75/25/0
PAV R1	204.63 ± 45.74	17.23 ± 12.56	0.63	95/4/0
PAV TR	157.50 ± 54.57	13.10 ± 12.93	0.49	87/12/1
PAV R2	194.94 ± 61.24	16.58 ± 14.24	0.61	92/7/1

*Note*. PBA = peak bag acceleration; PAV = peak angular velocity; R1 = Retention 1; TR = Transfer; R2 = Retention 2.

Regarding peak bag acceleration (Table 2), novices showed a small, possibly positive, effect of auditory biofeedback from baseline to Retention 1 and 2. The effect of dual tasking was possibly trivial. For experts’ peak bag acceleration, the effects of auditory biofeedback from baseline was likely positive for Retention 1 and 2. The effects of dual tasking were possibly trivial. The findings of both peak bag acceleration and angular velocity indicate that auditory biofeedback led to performance and technique improvements for the experts and only to performance improvements for novices.

## Discussion

Although biofeedback is not new, technology advances have now made movement-related externally focused biofeedback affordable for training interventions. In externally focused biofeedback, the movement performer receives feedback regarding the indirect external impact of their movements rather than direct internal feedback regarding bodily functioning. Using the boxing jab as the movement task, we aimed in this study to examine, in both boxing experts and novices, the effects of auditory biofeedback on the boxing jab performance (i.e., by providing feedback regarding bag acceleration from the jab) and on the boxing technique (i.e., trunk rotation) as measured from strategically-positioned inertial measurement units. We found that biofeedback with this external focus led to performance and technique improvements for the experts but only to performance improvements for the novices.

Though boxing jabs are a popular method of motor skill training for professional and recreational athletes, the movements of the body and the boxing jab have not been well-studied. Therefore, we cannot directly compare our findings with prior research. However, a plethora of studies of other movements have demonstrated that focusing attention externally, when encouraged via verbal instruction, can be effective at improving task performance on force production tasks (Porter et al., 2012; Vance et al., 2004; Zarghami et al., 2012). The findings from the current study replicate those benefits documented by earlier research on external focus of attention for force production tasks. The results are broadly in agreement with Roseman (2017) who found that Taekowndo kicking force-accuracy was greater in the distal external condition for both novices and expert martial artists. However, in their study the focus conditions did not affect peak activity of the hamstrings or quadriceps, and experts were reported to contact these muscles less than novices. Similar between group differences in technique execution were shown in the present study. For the experts, improvements in bag acceleration (10.28% and 7.35%) were accompanied by substantive increases in trunk rotation (17.23% and 16.58%) for Retention 1 and 2, respectively, giving empirical validation that their improvements in technique can be promoted indirectly via externally focused biofeedback. Perhaps surprisingly, there was no associated improvement in technique in the novices who demonstrated minimal changes in angular velocity of the trunk (-1.78% and 2.50%) for both Retention 1 and 2, respectively. Thus, their improved bag acceleration (6.88% and 9.67%) must have come from increased movements of the more distal body segments, such as the elbow (Stanley et al., 2018).

There is also a growing consensus that biofeedback can be effective in retraining body movements. For example, externally focused biofeedback has been shown to reduce tibia shock in runners by over 50% (Crowell & Davis, 2011) and measures of foot balance in army recruits by 17% (Sharma et al., 2014). The changes among our participants for the performance measures used in this study were lower, with changes for novices’ peak bag acceleration of 6.88% and 9.67%, and changes for experts of 10.28% and 7.35% for Retention 1 and 2, respectively. Of note, however these changes were consistently in the desired direction.

These data make clear that biofeedback with an external focus positively affected task performance for both novices and experts. Presumably, the participants used the additional biofeedback information to enhance the natural tactile and auditory signals that relate to the glove-bag impact. These findings indicate the relevance of external biofeedback using auditory cues to improve boxing jab performance. Indeed, it should be noted that the instructions given were both external and highly relevant to the task. Hitting a bag hard with a jab is meaningful. If instructions are external but irrelevant, then they may lose benefit (Roseman, 2017).

## Limitations and Directions for Future Research

Taken together, these findings align with studies with other participants and movement skills to illustrate the potency of external biofeedback in modifying movement variables. There are, however, some caveats to our approach. First, training adaptations may require a longer time period to permit participants to adjust to a new way of performing a movement. Notably, the jab involves a complex, sequential chain of events; changing trunk rotation will lead to a sequence of distal to proximal changes that may be difficult to achieve in such a short period of time. While we were able to infer short-term adaptations due to biofeedback in the present study among both novices and experts, the stronger performance improvement shown by experts is likely underpinned by these performers’ higher skill level and associated greater readiness to benefit from relatively brief external feedback training. However, this study did not provide a trial-by-trial analysis of the non feedback trials compared to the trials after the feedback was provided. In the future it will be important to study the longevity of these results over both trial-by-trial and extended retention periods in both experts and novices. Second, while the number of years of boxing experience can distinguish experts from novices, we had no other quantitative data with which to establish the experts’ supposed higher level of performance. It was therefore not possible to establish a definitive relationship between skill level and the performance-related variable. Finally, we did not employ a control group of novice or expert participants who received no external biofeedback on this task we cannot rule out that bag acceleration improvements observed were incremental gains over simple repeated trials of boxing jabs. The angular velocity data are robust against this possibility however, as the novices showed no increases across all blocks compared to the experts who did improve. Future studies should better control for this potential confound. Despite these limitations, however, we consider the experimental design sufficiently robust to enable us to assert effectiveness to externally-focused biofeedback as a means of improving performance on measures of task performance and technique.

## Conclusions

To reiterate we found that biofeedback is a potent technique for changing movement patterns and that external attentional focus in particular was effective in bringing about performance improvements among both novices and experts. These findings are in line with Wulf and Su (2007) and Ille et al. (2013). While our findings have direct relevance for improving boxing jab performance, the use of such biofeedback to improve movement patterns for injury avoidance, along with quick performance improvements for novices and movement consistency across a range of sports, is an important future application for this motor training method.

As far as we are aware, this is the first study to quantify the effect of externally focused biofeedback on measures related to both technique and performance. The results of our study demonstrate a positive effect of externally focused biofeedback on biomechanical variables associated with technique and performance. This study now paves the way for further investigations into the application of such biofeedback-based devices to support a range of sport, gaming and rehabilitation-focused training programs.
